# High Energy Storage Performance in Bi_0.46_Sr_0.06_Na_0.5_TiO_3_/CaTiO_3_ Relaxor Ferroelectric Ceramics

**DOI:** 10.3390/ma18214932

**Published:** 2025-10-28

**Authors:** Yangyang Zhang, Haizhou Guo, Shuyao Zhai, Liqin Yue, Juqin Zhang, Suxia He, Ruiling Fu, Chiyu Yin, Ling Zhang

**Affiliations:** 1 Henan Key Laboratory of Nanocomposites and Applications, Huanghe Science and Technology College, Zhengzhou 450000, China; yyzhang@hhstu.edu.cn (Y.Z.); mk314@126.com (J.Z.); hsx822@126.com (S.H.); 201106077@hhstu.edu.cn (R.F.); chiyu_yin@163.com (C.Y.); 2 Sensor R&D Center, Zhengzhou Winsen Electronics Technology Company Limited, Hanwei Electronics Group Corporation, Jinsuo Road, Zhengzhou 450000, China; guohz@winsensor.com; 3 College of Computer Science and Technology, Zhengzhou University of Light Industry, 136 Science Avenue, Zhengzhou 450001, China; andreasuya@163.com; 4 College of Mechanical and Electrical Engineering, Shihezi University, Shihezi 832061, China

**Keywords:** energy storage ceramics, (Bi_0.5_Na_0.5_)TiO_3_-based, frequency stability

## Abstract

(Bi_0.5_Na_0.5_)TiO_3_-based lead-free ferroelectric ceramics are among the most extensively researched energy storage materials today. In this paper, (1 − x)Bi_0.46_Sr_0.06_Na_0.5_TiO_3−x_CaTiO_3_ ceramics were synthesized through a solid-phase sintering method by synergistically adjusting CaTiO_3_ components after introducing Sr^2+^ at the A-site. The XRD patterns revealed that all samples formed a single perovskite solid solution, with the 111 and 200 peaks shifting to higher levels as the CaTiO_3_ increased, indicating a gradual decrease in cell volume. The SEM images exhibited dense crystals without any apparent porosity, which were formed by the different components of the ceramics. Through energy storage, dielectric, and charge–discharge performance tests, it was found that with a 10%mol CaTiO_3_ addition, the samples obtained a maximum breakdown field strength of 260 kV/cm and corresponding saturation polarization strength of 32.80 μC/cm^2^ and thereby exhibited a reversible energy storage density valued 3.52 J/cm^3^. In addition, the dielectric constant varied by less than 10% within the temperature range of 63.7 °C to 132.7 °C and presented good frequency (10–250 Hz) stability at 180 kV/cm. Moreover, the ceramics demonstrated a maximum current density reaching 349.58 A/cm^2^ and a maximum power density of 18.90 MW/cm^3^ for their charge–discharge performance, all of which makes them suitable for pulse system applications.

## 1. Introduction

Pulse power supplies have garnered significant attention owing to their critical applications in livelihood protection, industrial manufacturing, and the military sector. In recent years, dielectric capacitors (DCCs), which are core components of these systems, have emerged as a prominent research focus [1]. However, ceramic capacitors exhibit a relatively lower energy density compared to chemical cells and electrolytic capacitors, which remains a notable limitation [2]. The energy storage performance is primarily evaluated by effective energy storage density (*W*_rec_) and energy storage efficiency (*ŋ*) with the calculation formula as follows:

(1) $$W=\int_{0}^{P_{\max}}EdP$$

(2) $$W_{rec}=\int_{P_{r}}^{P_{\max}}EdP$$

(3) η=WrecW

where *E*, *P*_max_, and *P_r_* represent the applied electric field, saturation polarization, and remnant polarization, respectively. To achieve excellent energy storage capacity, it is essential to maximize *P*_max_ and *E* while minimizing *P_r_*. Relaxor ferroelectric ceramics, characterized by their elongated hysteresis loops, have thus become a focal point of our research [3]. Driven by the growing emphasis on environmental sustainability, lead-free ferroelectric materials have garnered significant attention among researchers [4]. Specifically, (Bi_0.5_Na_0.5_)TiO_3_-based (BNT) ceramics have emerged as a prominent research focus. The single-phase BNT ceramic exhibits a rectangular-shaped hysteresis loop, characterized by the advantage of a very high *P*_max_ but hindered by the drawbacks of a high *P_r_* and low breakdown field strength *E*. These limitations necessitate further improvements for practical applications [5].

According to a report by Muhammad R. et al., substituting Ca^2+^ at the A-site of BaTiO_3_ significantly impacts the ferroelectric properties of the matrix and enhances the relaxation behavior of the system [6]. Nayak S. et al [7]. developed (1 − x)(Ba_0.78_Ca_0.22_)TiO_3−x_BiScO_3_ ceramics. By incorporating Ca^2+^, which has a relatively smaller ionic radius, into the solid solution, they successfully reduced the cell volume. The dielectric temperature spectrum indicated that samples exhibited high activation energy, and the introduction of Ca^2+^ shifted the phase transition temperature to a lower range, thereby enhancing the electrical performance at room temperature. Dai Z. et al. introduced Sr^2+^ into (K_0.5_Na_0.5_)NbO_3_ by adding (Sr_0.5_Nb_0.5_)O_3_, resulting in unequal substitution which significantly boosted the *W_r_* to a high level [8]. In conclusion, several researchers have successfully enhanced the relaxation properties of perovskite ceramics by incorporating Ca^2+^ and Sr^2+^ into other systems. This approach has resulted in an increase in the breakdown field strength *E* and a substantial reduction in *P_r_*, providing valuable insights and inspiration for this study.

In this study, (1 − x)Bi_0.46_Sr_0.06_Na_0.5_TiO_3−x_CaTiO_3_ ceramics were synthesized. This was achieved by introducing Sr^2+^ at the A-site and incorporating CaTiO_3_ components. The structural, morphological, and functional properties of BSNT-CT ceramics, including XRD patterns, SEM microstructures, energy storage performance, dielectric behavior, and charge–discharge performance, were systematically investigated.

## 2. Materials and Methods

### Preparation of BSNT-xCT Samples

The classical solid-phase method was used to prepare (1 − x)Bi_0.46_Sr_0.06_Na_0.5_TiO_3−x_CaTiO_3_ (x = 0, 0.04, 0.06, 0.08, 0.10, and 0.12) (BSNT-*x*CT) ceramics. The required reagents were calculated according to their recipes, dried, and weighed: Bi_2_O_3_ (99.9%, Aladdin, Shanghai, China), Na_2_CO_3_ (99.5%, Aladdin), SrCO_3_ (99.95%, Aladdin), CaTiO_3_ (99.5%, Aladdin), and TiO_2_ (99%, Aladdin). After ball milling using ethanol and zirconia balls as the medium for 6 h and drying, BSNT and CT were obtained by pre-sintering at 700 °C and 800 °C for 3h, respectively, with a heating rate of 150 °C/h. After secondary ball milling and drying, a series of ceramics were prepared and pressed into rough embryos with a 12 mm diameter and 1.0 mm thickness at 300 Mpa. The compacts were finally sintered at 1000 °C for 3 h with a heating rate of 150 °C/h, followed by furnace cooling. To compensate for component volatilization during high-temperature sintering, each green body needed to be placed in a sealed crucible and covered with powder of the same composition on its surface.

The crystal structure was characterized by X-ray diffraction (XRD) using a Bruker D8 Advance diffractometer (Panalytical B.V., Almelo, The Netherlands) with Cu Kα radiation (λ = 0.15418 nm), while the surface particle images were obtained using a scanning electron microscope (SU8010, Hitachi Ltd., Tokyo, Japan) operated at an accelerating voltage of 20.0 kV. Silver electrodes with a diameter of 3 mm were fabricated on the ceramic pellets by screen-printing a silver paste, followed by a high-temperature curing process. Subsequently, the *P*-*E* loops of the samples were measured using a ferroelectric analysis system (LC II-100V, Radiant Technologies Inc., Burbank, CA, USA) in double hysteresis loop mode; the charge/discharge performance was evaluated with a capacitive charging and discharging testing system (CDTS-10, JYJS Technology Co., Ltd., Wuhan, China); and the dielectric temperature spectra were obtained using a dielectric temperature spectrometer (YHJW-1000, Yunhe Star Science and Technology Co., Ltd., Wuhan, China) equipped with an automatic temperature-controlled heating furnace.

## 3. Results

**Figure 1:**
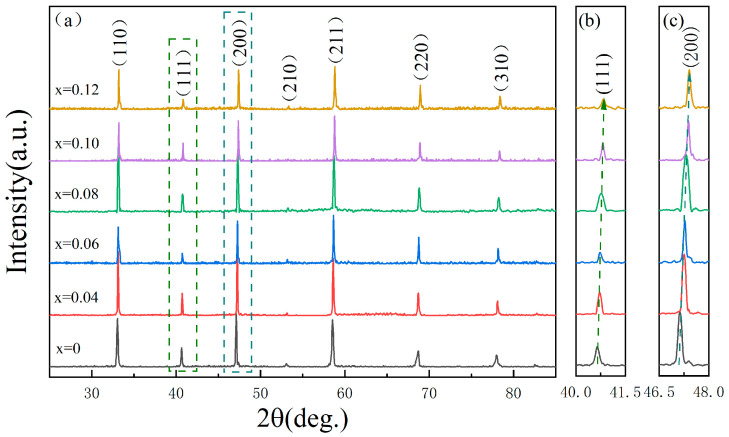
(**a**) XRD spectrum of BSNT-*x*CT ceramics, (**b**) the enlarged image of (111) diffraction peaks and (**c**) the enlarged image of (200) diffraction peaks.

Figure 1 displays the XRD patterns of BSNT-*x*CT samples. Figure 1a compares the XRD spectra of systems with varying CT doping levels. By referring to the standard diffraction datas from the JCPDS-ICDD Powder Diffraction Database, it is evident that all doping ratios of the ceramics exhibit a high-purity perovskite structure. This signifies that the BSNT and CT components form a good BSNT-*x*CT solid solution within the specified chemical composition range. Figure 1b,c illustrates the enlarged versions of the (111) and (200) peaks for systems with differing doping levels. It clearly demonstrates that, as CT components increase, the corresponding peaks gradually shift towards higher angles. This phenomenon indicates a reduction in cell volume, which can be attributed to the introduction of Ca^2+^ (1.34 Å), whose ionic radius is smaller than that of Bi^3+^ (1.38 Å) and Sr^2+^ (1.44 Å) [9]. Moreover, it is noteworthy that when the CT content exceeds 8 mol%, the diffraction peak intensity exhibits a discernible decrease, which indicates a substantial escalation in unit-cell distortion, suggesting a probable significant reduction in grain size.

**Figure 2:**
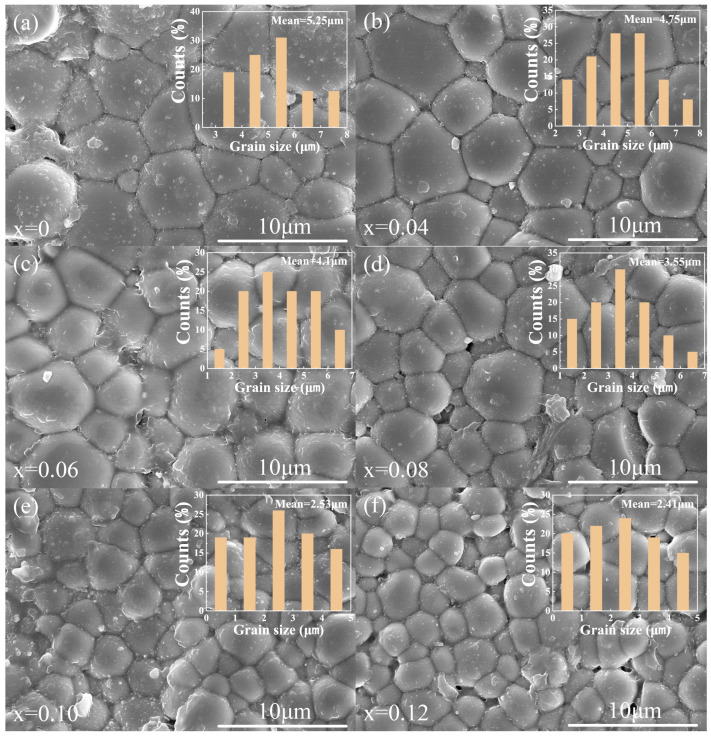
(**a**–**f**) The surface SEM images of various BSNT-*x*CT ceramics.

**Figure 3:**
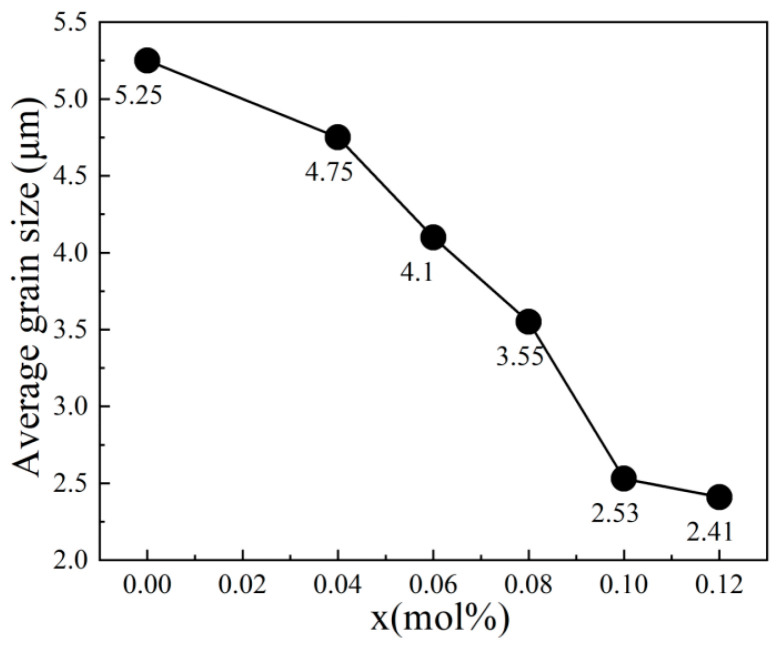
Variation curve of mean grain size with CT content of BSNT-*x*CT ceramics.

Figure 2a–f present the surface SEM images and grain distribution of each component of the BSNT-*x*CT ceramics. The images reveal that all the BSNT-*x*CT ceramic components exhibit dense sintering without evident pores or second phases. Additionally, statistical analysis presented in the inset demonstrates a consistent trend of decreasing grain size along with improved size uniformity. This phenomenon can be attributed to the increased structural stress induced by lattice distortion following component compositing, which effectively suppresses grain growth. Figure 3 illustrates the correlation between the average grain size (AGS) and varying CT contents. Therein, the AGS was determined by averaging the diameters of multiple grains, while the size of an individual grain was taken as its equivalent circular diameter. As the CT content increased, the AGS progressively diminished, which helped to create a relatively larger grain boundary proportion, thereby enhancing the breakdown electric field strength and consequently improving the energy storage capacity [10].

**Figure 4:**
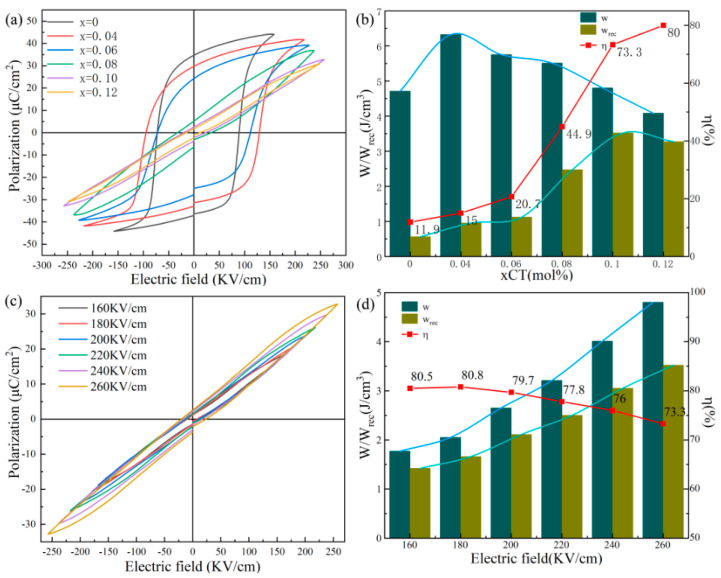
Different components of BSNT-*x*CT ceramics: (**a**) *P*-*E* curves and (**b**) *W*, *W*_rec_, and *ŋ*, 0.9BSNT-0.1CT samples in different applied fields, (**c**) *P*-*E* curves, and (**d**) *W*, *W*_rec_, and *ŋ*.

**Figure 5:**
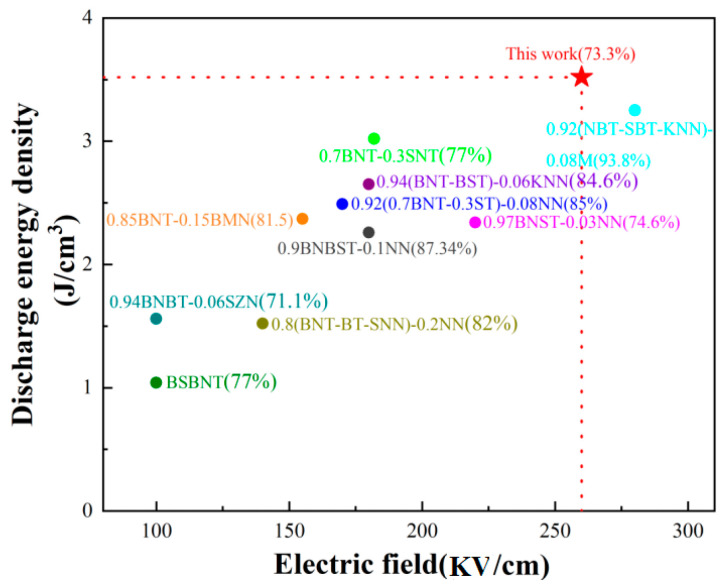
Comparison of *W*_rec_ and *ŋ* of this study with other reported studies [11,12,13,14,15,16,17,18].

Figure 4a gives the bipolar *P*-*E* curves of BSNT-*x*CT samples under maximum breakdown field strength with a test frequency of 10 Hz, which clearly demonstrates that the addition of the CT component effectively reduced the ferroelectricity of BNST-based ceramics and enhanced the relaxation performance. When the CT content increased, the *P_r_* decreased rapidly, whereas the *P*_max_ of the ceramics containing 10 mol% CT reached a high of 32.80 μC/cm^2^. Larger *P*_max_ and lower *P_r_* are advantageous for enhancing energy storage capacity, as exemplified in Figure 4b [9]. This improvement can be attributed to the introduction of Ca^2+^, which reduces the cell volume, densifies the ceramic, and subsequently enhances *W*_rec_ and *ŋ*. The test data reveals that, for x = 0.1, the 0.9BSNT-0.1CT ceramic exhibits a *W*_rec_ valued at 3.52 J/cm^3^ and a *ŋ* of 73.3% under 260 kV/cm, which makes this ceramic stand out compared to most recent reports of the same ceramic systems as shown in Figure 5 [11,12,13,14,15,16,17,18].

**Figure 6:**
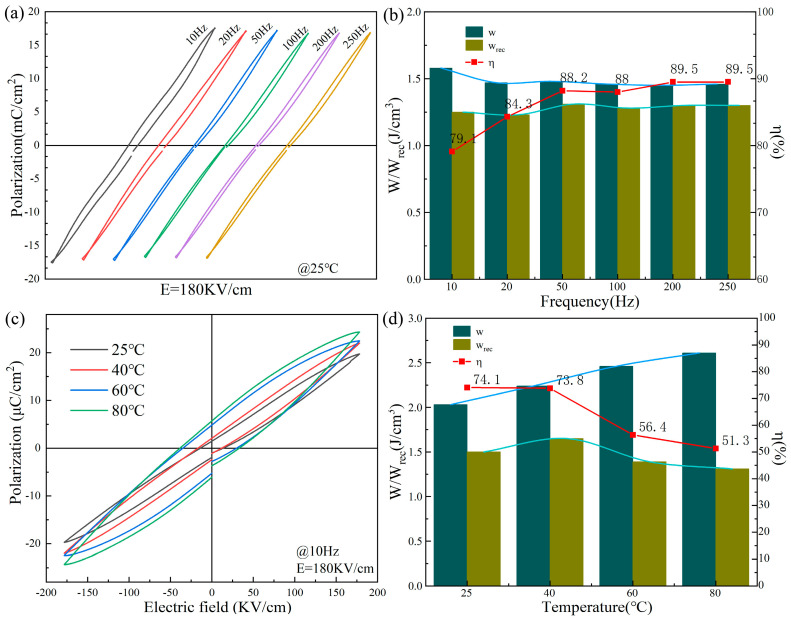
0.9BSNT-0.1CT samples: (**a**) *P*-*E* curves and (**b**) *W*_rec_, *W*, and *ŋ* at 10–250 Hz, (**c**) *P*-*E* curves and (**d**) *W*_rec_, *W*, and *ŋ* at 25–80 °C.

Given that pulse energy storage capacitors frequently operate at variable frequencies and under high energy consumption conditions, we conducted tests on features under various temperature and frequency conditions [19]. Figure 6a gives the *P*-*E* curves obtained for the 0.9BSNT-0.1CT ceramics tested at various frequencies. All curves show an elongated shape, and the *P*_max_ slightly decreases as the frequency rises. The histogram in Figure 6b shows that the *W*_rec_ fluctuates in the range of 1.25–1.3 J/cm^3^ with *ŋ* is in the range of 79.1–89.5% at different frequencies. Figure 6c,d show the *P*-*E* curves and calculation results at various temperatures, with the *W*_rec_ fluctuating in the range of 1.31–1.50 J/cm^3^ while *ŋ* is in the range of 51.3–74.1% at 25–80 °C, which indicates that 0.9BSNT-0.1CT ceramics possess good temperature and frequency stability in a certain range.

**Figure 7:**
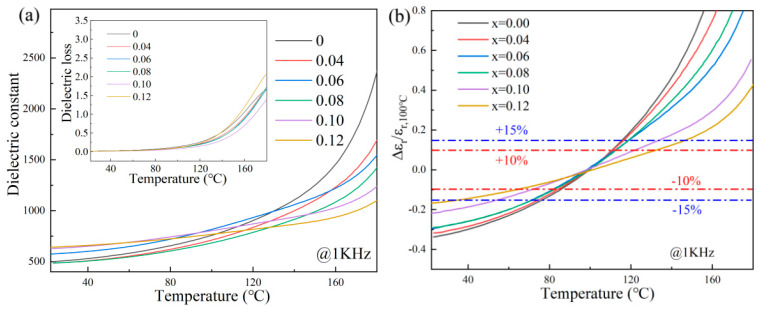
BSNT-*x*CT ceramics at 1 kHz (**a**) Comparison of the curves of *ε_r_* and tanδ, (**b**) Δ*ε_r_*/*ε*_*r*,100°C_ Variation range curve with temperature.

The *ε_r_* and tanδ variation curves at different temperatures for BSNT-CT ceramics are presented in Figure 7a,b. Since the testing temperature was below the Curie point, the dielectric constant increased with rising temperatures due to enhanced ionic displacement polarization. Within the tested temperature range of 20–180 °C, all compositions exhibited broadened and flattened dielectric constant curves with pronounced frequency dispersion. Furthermore, the incorporation of CT content was observed to progressively suppress the dielectric constant. To quantitatively assess the impact of CT components on the dielectric characteristics of BSNT-*x*CT samples, Figure 7a gives the variation curves of *ε_r_* and tanδ at different temperatures for BSNT-*x*CT ceramics. As the temperature changes from 20 °C to 120 °C, the ceramic dielectric constant (*ε_r_*) curves of different compositions maintain parallel alignment with similar variation trends, exhibiting a mutual deviation Δ*ε_r_*/*ε*_*r*,*x*=0_ < ±15%. Meanwhile, the dielectric loss tanδ remains below 0.35, indicating a low level. Figure 7b displays the test data collected at 1 kHz as a sample, using *ε_r_* at 100 °C as the reference. The variation curve of Δ*ε_r_*/*ε*_*r*,100°C_ with temperature is observed. Calculations reveal that as the CT content increases, the temperature range where Δ*ε_r_*/*ε*_*r*,100°C_ < ±10% gradually widens, shifting from 83 to 112 °C at *x* = 0 to 63.7–132.7 °C at *x* = 0.12. This suggests that the solid solution, when synthesized effectively, enhances the dielectric temperature stability [20].

**Figure 8:**
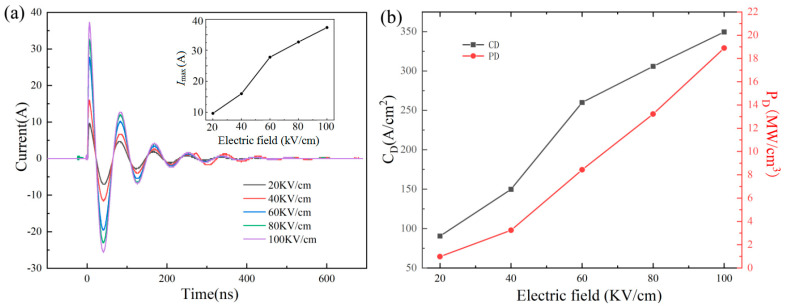
0.9BSNT-0.1CT ceramics: (**a**) underdamped discharge waveforms under various electric fields, inserted as *I*_max_; (**b**) relationship between *C*_D_ and *P*_D_ and field strength.

**Figure 9:**
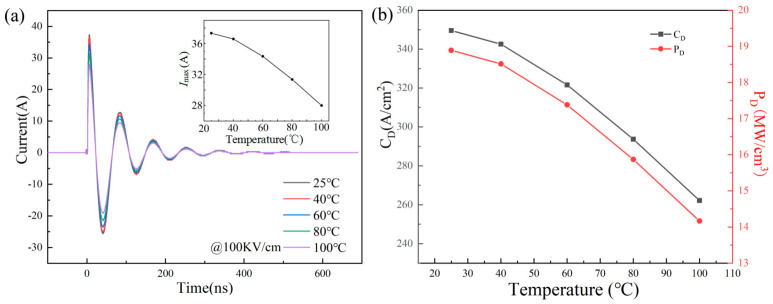
0.9BSNT-0.1CT ceramics: (**a**) underdamped discharge waveforms at different temperatures, inserted as *I*_max_; (**b**) relationship between *C*_D_ and *P*_D_ and temperature.

To verify the capacitor performance, the charge and discharge performance also needs to be tested. Figure 8a illustrates the waveform curves of current versus time for underdamped discharge of 0.9BSNT-0.1CT ceramics under various applied fields. The curves at varying voltages tend towards zero after a few cycles, and the increasing current peak from 9.7 A to 37.3 A is directly proportional to the external electric field. Figure 8b calculates the current density *C*_D_ (*C*_D_ = *I*_max_/*S*) and power density *P*_D_ (*P*_D_ = *I*_max_*E*/2*S*), where *S* is the area of the electrode used for testing. The line graph demonstrates that both *C*_D_ and *P*_D_ increase as *E* increases. The *I*_max_ of 37.3A is achieved at a maximum of 100 kV/cm, with a *C*_D_ of 349.58 A/cm^2^ and a *P*_D_ of 18.90 MW/cm^3^. Figure 9a,b show the underdamped discharge waveforms for temperature variations (25–100 °C). Between 25 °C and 60 °C, *I*_max_, *C*_D_, and *P*_D_ exhibit good stability with less than 10% change. However, as the temperature exceeds 60 °C, there is an increase in decay, which may be attributed to the rise in sample resistance at higher temperatures.

## 4. Conclusions

In this paper, a comprehensive investigation of BSNT-*x*CT ceramics was conducted on their microstructure, energy storage capabilities, dielectric properties, and charge–discharge performance. As the *x* value increases, the ceramic cell size decreases, density increases, and BSNT and CT components form a well-integrated solid solution. At *x* = 0.1, the maximum breakdown field strength reaches 260 kV/cm, with a corresponding maximum polarization of 32.80 μC/cm^2^. Calculations reveal that the 0.9BSNT-0.1CT ceramic obtains a *W*_rec_ value of 3.52 J/cm^3^ and a *ŋ* of 73.3%. Furthermore, the dielectric constant exhibits minimal variation (less than 10%) across a broad temperature spectrum from 63.7 °C to 132.7 °C. The *C*_D_ stands at 349.58 A/cm^2^, while the *P*_D_ is 18.90 MW/cm^3^. Additionally, the charge/discharge performance demonstrates minimal variation (less than 10%) within the temperature range of 25 °C to 60 °C. These findings suggest that this series of ceramics holds significant promise for applications in pulsed systems.

## Data Availability

The original contributions presented in this study are included in the article. Further inquiries can be directed to the corresponding authors.
